# Associated Liver Partition and Portal Vein Ligation (ALPPS) vs Selective Portal Vein Ligation (PVL) for Staged Hepatectomy in a Rat Model. Similar Regenerative Response?

**DOI:** 10.1371/journal.pone.0144096

**Published:** 2015-12-02

**Authors:** Rocío García-Pérez, Beatriz Revilla-Nuin, Carlos M. Martínez, Angel Bernabé-García, Alberto Baroja Mazo, Pascual Parrilla Paricio

**Affiliations:** 1 Department of Surgery, Experimental Surgery Unit, IMIB-LAIB Research Center, El Palmar (Murcia), Spain; 2 CIBERehd, Barcelona, Spain; University of Navarra School of Medicine and Center for Applied Medical Research (CIMA), SPAIN

## Abstract

Associated liver partition and portal vein ligation for staged hepatectomy (ALPPS) is a two-stage hepatectomy technique which can be associated with a hypertrophic stimulus on the future liver remnant (FLR) stronger than other techniques–such as portal vein ligation (PVL). However, the reason of such hypertrophy is still unclear, but it is suggested that liver transection combined with portal vein ligation (ALPPS) during the first stage of this technique may play a key role. The aim of this study is to compare the hypertrophic stimulus on the FLR and the clinical changes associated with both ALPPS and PVL in a rat surgical model. For this purpose, three groups of SD rats were used, namely ALPPS (n = 30), PVL (n = 30) and sham-treated (n = 30). The second stage of ALPPS (hepatectomy of the atrophic lobes), was performed at day 8. Blood and FLR samples were collected at 1, 24, 48 hours, 8 days and 12 weeks after the surgeries. ALPPS provoked a greater degree of hypertrophy of the FLR than the PVL at 48 hours and 8 days (p<0.05). The molecular pattern was also different, with the highest expression of IL-1β at 24h, IL-6 at 8 days, and HGF and TNF-α at 48 hours and 8 days (p<0.05). ALPPS also brought about a mild proliferative stimulus at 12 weeks, with a higher expression of HGF and TGF-β (p<0.05) than PVL. Clinically, ALPPS caused a significant liver damage during the first 48 hours, with a recovery of liver function at day 8. In conclusion, ALPPS seems to induce higher functional hypertrophy on the FLR than PVL at day 8. Such regenerative response seems to be leaded by a complex interaction between pro-mitogenic (IL-6, HGF, TNF-α) and antiproliferative (IL1-β and TGF-β) cytokines.

## Introduction

The two-stage hepatectomy is a surgical strategy for patients with unresectable liver metastases or primary malignancies. The aim of these techniques is to remove all metastases in the FLR (which has insufficient volume to maintain the liver functionality) and induce its hypertrophy [1]. Thus, the success of the second procedure depends on the size and function of the FLR [2]. For a normal hepatic function, a FLR of 25% is usually considered sufficient to maintain hepatic homeostasis after the resection, but this percentage can be increased up to 40% in those patients with hepatic dysfunction or early liver injury [3]. Several strategies have been used for the induction of such hypertrophy for the second stage associating portal vein embolization (PVE) or ligation (PVL), but there are around 40% of cases of failure due to tumor progression during the hypertrophic stimulus (4–8 weeks) or ineffective volume after the application of these techniques [4–7].

The biological mechanisms of regeneration associated with these techniques seem to be different from conventional hepatectomy, and there is still controversy about which is the best technique (associating PVE or PVL) to cause such hypertrophy of the FLR [4–5, 8–11]. Associated liver partition and portal vein ligation for staged hepatectomy (ALPPS), is a surgical procedure which combines a PVL and an *in situ* transection of the remaining liver affected by metastases that has recently emerged as a real approach to cause a higher hypertrophy of the FLR in comparison with regional portal occlusion techniques, in a shorter period of time (9 days vs. 4 weeks). Thus, the ALPPS technique seems to be a good option in those scenarios where hypertrophic stimuli brought about by portal occlusion techniques are not expected to induce enough FLR in an acceptable waiting period to proceed to the second hepatectomy stage [12–14]. The first step of the ALPPS includes selective ligation of the tributary branches of the portal vein and transection of the affected lobe. These combined techniques seem to cause a significant increase in liver hypertrophy, although this increase might not reflect functional capacity, and the role of the procedure or the surgical trauma caused by the transection in such hypertrophy remains unclear [13]. Previous experimental murine models of ALPPS pointed a role of pro-inflammatory factors, like IL-6 and TNF-α in the regenerative process induced by the transection procedure [15], but the role of other pro-inflammatory cytokines (like IL1-β), or grown factors (like TGF-β) remains unclear. The aim of this study is to explore the morphometric, immunohistopathologic and molecular changes of the FLR and the clinical effects that the ligation of the tributaries of the portal vein simultaneously with a hepatic transection bring about in a rat surgical experimental model.

## Material and Methods

### Animals and experimental design

The experiments were performed on male Sprague-Dawley rats weighing 280–300 g. (Harlan Iberica, Barcelona, Spain). The animals were kept in an SPF environment at the animal facilities of Universidad de Murcia with food, water *ad libitum*, and light and dark cycles until the surgical procedures. The three groups comprised a selective PVL (n = 30), ALPPS (n = 30) and the sham-operated (control, n = 30). The same person performed all the surgeries. Groups of 6 animals were sacrificed at 1, 24 and 48 hours, 8 days (first stage of the ALPPS) and 11 weeks after the second stage of the ALPPS (hepatectomy of the atrophic lobes). This study was carried out in strict accordance with the recommendations stated by the European Union about protection of animals used for scientific purposes (Directive 2010/63/EU). The protocol was approved by the Committee on the Ethics of Animal Experiments of the Universidad de Murcia (Permission Number: A1320140705). Animals were managed only by specialized personnel from the same institution.

### Surgical procedures

**Fig 1:**
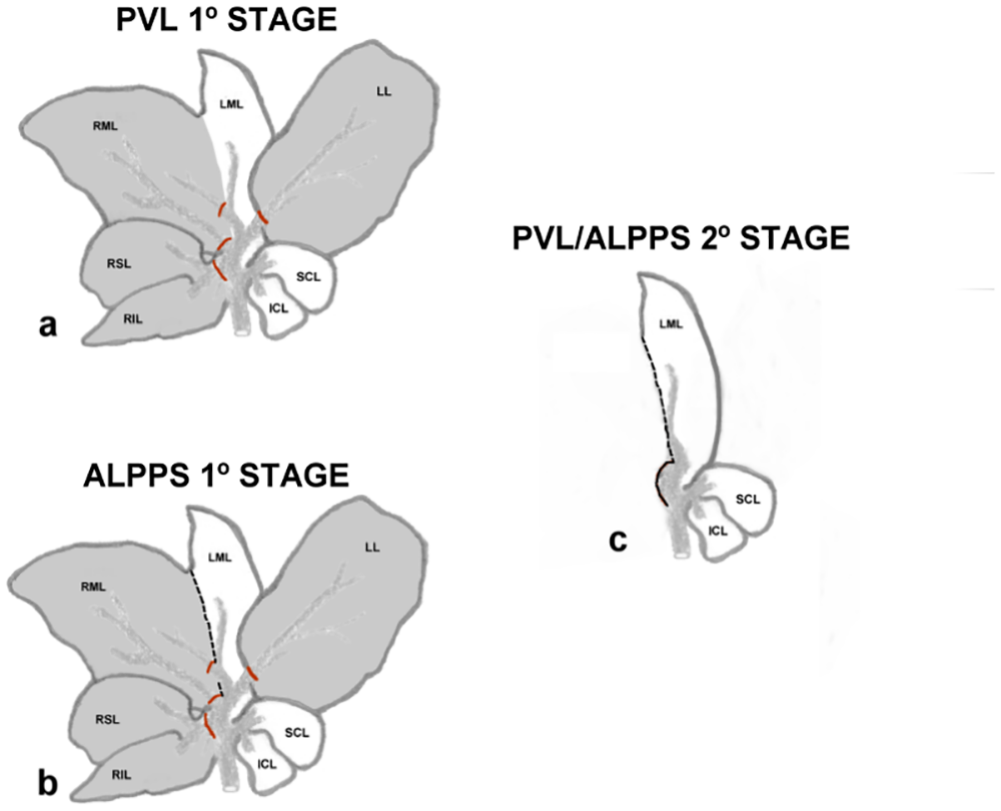
Schematic representation of the PVL and ALPPS procedures on the rat liver. (**a**) For the PVL, selective ligation (dark areas and red lines) of the portal flow was assessed on the right superior (RSL), right inferior (RIL), left (LL), and the right portion of the middle lobes (RML), while the left portion of the middle lobe (LML), superior caudate (SCL), and inferior caudate (ICL) were not ligated. (**b**) Regarding the first stage of the ALPPS procedure, a complete *in situ* transection (black line) of the median lobe was also performed after the PVL. (**c**) After 8 days, the second stage comprised the excision of the ligated/atrophic lobes.

Previous studies suggest that the basis of the rapid hypertrophy of the FLR associated with the ALPPS might be related to the physical interruption of the collateral irrigation between lobes caused by their *in situ* transection [13]. Thus, the first step taken was to study the lobar anatomic features of the rat liver which has no parenchymal bridges between lobes [16], to determine which one is suitable to perform the transection procedure in a similar way to its human application. As in previous studies [15], the medium lobe was chosen to develop the procedure because it consists of two portions: left and right portions, which are separated by a fissure [17]. It was observed that the selective portal occlusion of the right superior, right inferior, and right portion of medium lobes and caudate lobes (Fig 1a) provoked the suppression of vascular flow without leading the animals to a fatal hepatic failure. For the PVL procedure, the technique previously described was implemented [18].

To put it briefly, after midline laparotomy, all hepatic ligaments were dissected so as to assure the correct management of the hepatic lobes. After microdissection of hepatic artery and portal vein, tributary portal branches, which irrigate the previously named lobes (right superior, right inferior, left and right portion of medium lobes), were ligated by using 4/0 silk. To avoid the possible ligation of hepatic arteries or biliary ducts, all surgeries were performed at 16x magnification by using a Zeiss OPMI 6 surgical microscope (Carl Zeiss, Jenna, Germany). Additionally, the correct assessment of the technique was undertaken by histopathologic examination. For the first stage for the ALPPS procedure, after the PVL of the same lobes, the medium lobe was completely split (Fig 1b) by using a bipolar coagulator (B. Braun). Finally, the midline laparotomy was closed in two layers by using an absorbable suture (polyglicolic acid) and 3/0 silk. All the animals were under observation on a daily basis, and in order to avoid suffering, dosages of subcutaneous buprenorphine dose were administered after the procedure (0.05mg/kg every12 hours for the first 72 hours, then one dose/24 hours to day 5). All the animals were kept in an SPF and dark environment for recovery and observation.

**Fig 2:**
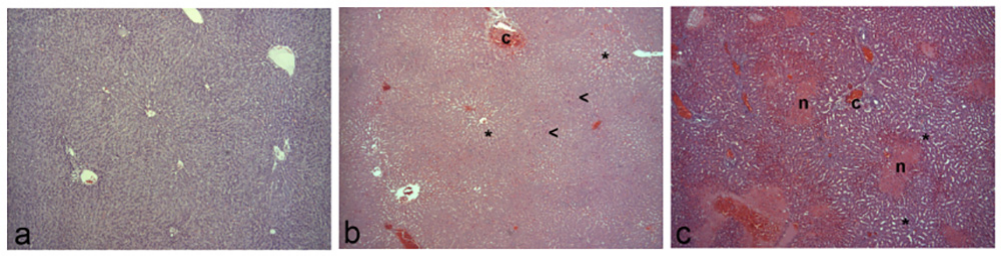
Representative microscopic images of control, PVL, and ALPPS atrophic lobes at 24 HPS. In (**a**) control liver, areas of congestion and sinusoid dilation (asterisks) could be observed. (**b**) In PVL atrophic lobes, scattered areas of degenerative hepatocytes (arrow heads) can be evidenced. (**c**) In ALPPS ones pericentrilobular areas of necrosis (n) surrounding the centrolobular vein (c) were present. (a,b,c): hematoxylin and eosin stain. X50.

The second stage of the ALPPS and PVL procedures (hepatectomy of the atrophic lobes) comprised the excision of the ligated (atrophic) lobes at day 8 days of the first stage (Fig 2c). After the re-opening of the midline laparotomy, all ligated/atrophic lobes were removed, closing the abdominal wall again by layers as described above. Then, the same routine to control animal suffering for the first stage was followed again for the second stage.

### Analysis of morbidity/mortality associated with the surgical procedure

In order to determine the parameter of morbidity/mortality, all the animals were examined twice a day during the first 72 hours after surgery and then on a daily basis during 12 weeks after the procedure. The clinical symptoms were evaluated by specialized staff.

### Liver morphometry

All the animals were sacrificed by using a mixture of 4% isofluorane and saturated carbon dioxide atmosphere, according to recommendations in the same Directive (Directive 2010/63/EU). Once the liver was explanted, the FLR was carefully dissected and weighed by using a standard laboratory scale (Adam, Milton Keynes, UK). After weighing, the volume was measured by fluid displacement as previously described [19]. All morphometric measurements were repeated three times to assure the reproducibility of the results.

### Histology and immunohistochemistry

For histopathologic examination, samples of FLR and atrophic lobes were fixed in 4% buffered formalin (Panreac Quimica, Madrid, Spain) for 24 hours, processed, paraffin-embedded, 3μm-sectioned and stained with a standard hematoxylin and eosin (H&E) stain. The mitotic index (MI) was considered as the average of mitoses counted in 10 random-high-power fields (HPF). Sections were also stained for Ki-67 expression (monoclonal rabbit anti-Ki-67 antibody, Master Diagnostica, Granada, Spain), endothelial cells (monoclonal mouse anti-CD31 (clone TL3A12), Abcam, UK), and Kupffer cells (monoclonal mouse anti-CD68 (clone ED1), Serotec, UK) using a standard ABC commercial kit in an automated immunostaining system (DakoEnVision and DakoAutostainer Plus, Dako, USA) following the manufacturer´s recommendations. The proliferation index (PI) was established as the average percentage of nuclear Ki-67 positive hepatocytes in 10 HPF. The Kupffer cell rate was considered as the average of positive cells counted in 10 HPF. A blinded microscopic analysis was performed by using a modular light microscope with color camera and specialized digital software (Zeiss Axio Scope A1, Carl Zeiss, Jenna, Germany).

### Molecular biology analysis

For molecular biology, samples from the FLR were immersed in RNAlater (Sigma, Madrid, Spain) for 24 hours at 6–8°C, and storaged at -80°C until use. RNA was then extracted from liver tissue stored in RNAlater using the RNeasy^®^ mini Kit (Qiagen, Germany), with on-column DNase I (Qiagen) digestion, following the manufacturer's instructions. The extracted RNA was quantified by absorbance at 260 nm and its purity was evaluated by the absorbance ratio at 260/280 nm with a NanoDrop -2000 spectrophotometer (Fisher Scientific, Madrid, Spain). cDNA was prepared using iScriptcDNA Synthesis kit (BIO-RAD Laboratories Inc.) according to the manufacturer protocol. mRNA levels were measured in duplicate with reagents from the SYBR Premix Ex Taq (TliRNaseH Plus) kit (Takara Bio, Inc.) and Quantitect primer Assay (Qiagen, Germany) in MyIQ5 system (BioRad Laboratories, Inc.). Negative control reactions without RT reaction and template were also performed. A melting curve analysis was performed in each run to ensure specificity of the primers. The mRNA expression data were normalized with respect to levels of GADPH (Quantitec Primer Assays, Qiagen, Germany). The cycle number at which the real-time PCR reaction reached an arbitrarily determinate threshold (CT) was recorded for both the mRNAs and GAPDH, and the relative amount of mRNA to GAPDH was described as 2^-ΔCT^ where ΔCT = (CT mRNA- CT GAPDH). Primers for HGF, TGF-β, IL6, IL1-β, TNF-α and TGF-α were purchased from Quiagen.

### Blood and clotting investigations

For clinical investigations, blood samples were obtained by cardiac puncture in a dried tube (BD Vacutainer^®^, Madrid, Spain) and centrifuged at 2600 rpm for 5 minutes. An automatic serum analysis for aspartate (AST) and alanine (ALT) aminotransferases and total bilirubin was performed using a serum multiple biochemical analyzer (Roche/Hitachi Modular Pre-Analytic Plus System, Roche Diagnostics, Indianapolis, USA). For blood clotting investigations, tubes with 0.1% citrate buffer (BD) were used for sampling and centrifuged at 3000 rpm for 10 minutes. The serum was then extracted and stored at -20°C until use. The levels of prothrombin (PT), activated partial thromboplastin time (APTT) and fibrinogen were measured using a serum multiple hemostasis testing system (ACLTop-300 CTS, Instrumentation Laboratories, Barcelona, Spain). All the results were expressed in IU/L.

### Statistics

Statistical analysis was performed using a software package (GraphPad Prism, Ver. 6, GraphPad Software Inc., California, USA). Statistical differences between groups were assessed by a two-tailed Mann-Whitney non-parametric test. A p-value of <0.05 was considered as significant. The size of groups (n = 6) was calculated to establish a statistic power of 83.4% (G*Power, Ver. 3.1.9.2), expecting medium-high differences between medians based on previous studies on FLR hypertrophy by these techniques [12–15]. All the numerical data are expressed as the ±standard deviation of the median.

## Results

### Morbidity/mortality associated with the surgical procedures

As regards the results of the study, all the animals involved showed moderate to severe clinical morbidity immediately after surgery. Clinical symptoms include apathy, anorexia, rough hair, and lethargy. These symptoms were noted within the first 72 hours after surgery. After that moment, the symptoms gradually reduced until the complete recovery of the animals at day 7–8. No other co-morbidities were observed during the 12 weeks of the study. Regarding mortality rates, no deaths were registered during the PVL procedure, a result which contrasts with the ALPPS procedure, in which a 17% of mortality (5/30 animals) was registered within the first 48 hours after the procedure.

### Histopathological assessment of PVL and ALLPS

The most common procedure to study liver regeneration is based on a surgical removal of the 2/3 (70%) of the liver [19]. In our model, the average weight of the atrophic lobes was 9.4±0.40, which represents the 78.72% of the total liver weight in sham-treated animals (11.94±1.28gr, n = 6). The main microscopic features at 24 HPS on the atrophic lobes were periportal congestion, sinusoid dilation and scattered areas of degenerative hepatocytes (Fig 2b and 2c) and scattered areas of centrilobular necrosis in the ALPPS group (Fig 2c).

### Morphometric changes on the PVL and ALPPS FLR

**Fig 3:**
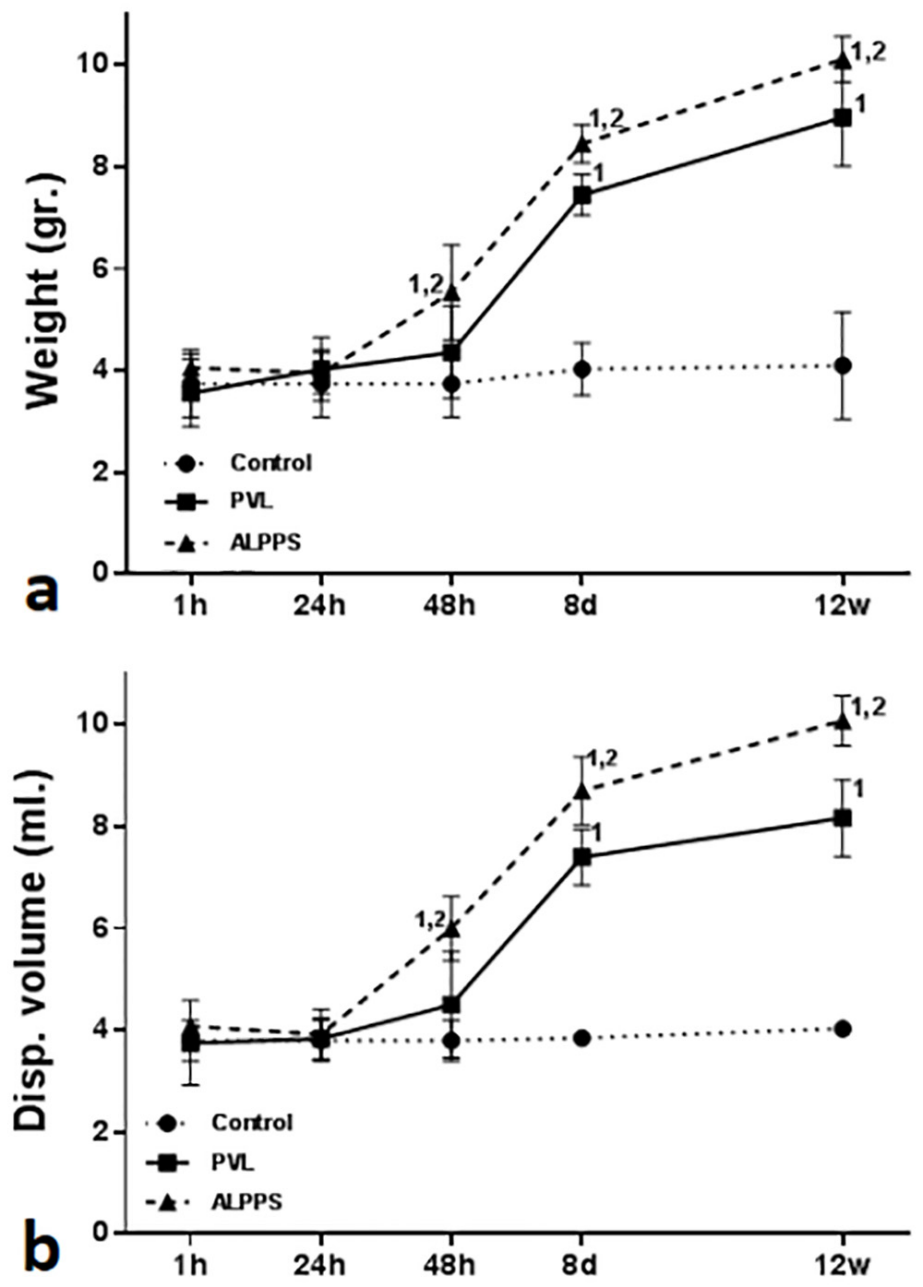
Morphometric evolution of weight and displaced volume of the controls, PVL and ALPPS FLR. (**a**) There is an increasing of the weight of PVL and ALPPS FLR from 48 hours post-surgery, but significantly higher in this last group. (**b**) Regarding the evaluation of the displacement volume the same results are observed. Mann-Whitney U-test (n = 6 animals per group). The numbers (1, 2) indicate significant differences of the considered group in comparison with control (1) and PVL (2) groups, respectively. A p value of <0.05 was considered as significant.

Morphometric measurements showed that the hepatic transection brought about a significant increase in both weight (Fig 3a) and volume (Fig 3b) of the FLR during the first 48 hours post-surgery and continued during all the experience (p<0.05) in comparison with the PVL alone.

### Immunohistopathologic features of the PVL and the ALPPS FLR

**Fig 4:**
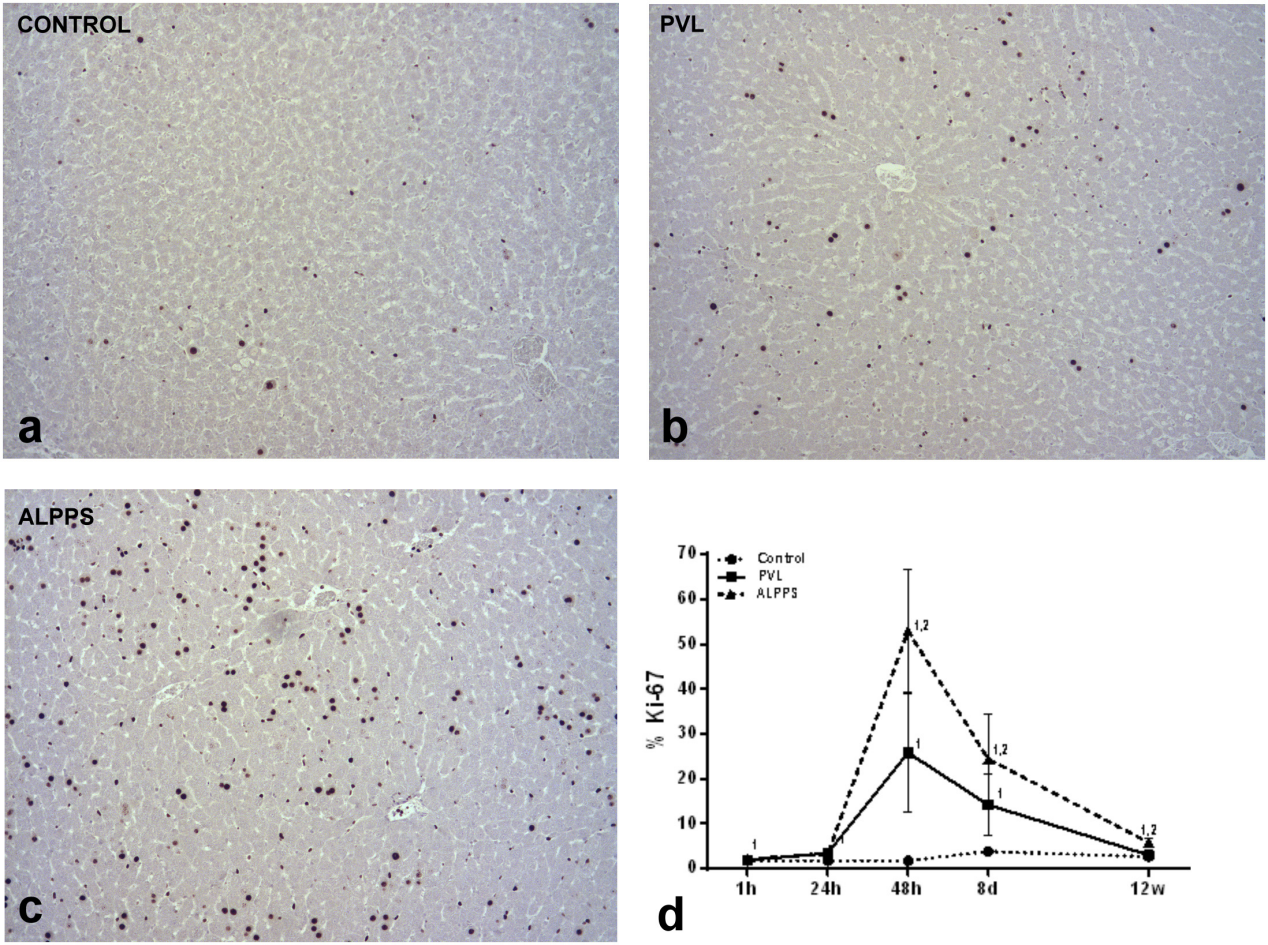
Representative microscopic images at day 8 post surgery, and temporal evolution of Ki-67 immunohistochemical expression in control, PVL and in ALPPS FLR. (**a**) In control liver, there is a little expression of Ki-67. (**b**) In PVL, there is an increase in Ki-67 in comparison with controls. (**c**) In ALPPS groups, there is a significantly increased expression in comparison with PVL at day 8. (**d**) The temporal evolution of Ki-67 expression showed a significant increase in expression of Ki-67 of ALPPS FLR, with a peak of expression at 48 hours. Mann-Whitney U-test (n = 6 animals per group). The numbers (1, 2) indicate significant differences of the considered group in comparison with control (1) and PVL (2) groups, respectively. A p value of <0.05 was considered as significant.

The examination of the FLR showed that both techniques provoked a slight congestion and sinusoid dilation during the first 48 hours. Microscopically, there were no alterations at 8 days, but at 12 weeks slight biliary hyperplasia and several connective adherences could be observed in both groups. Mitotic figures were detected at 48 hours and 8 days, with higher MI in the ALPPS FLR than in the PVL (3.0±0.74 and 0.61±0.07 vs. 1.1±0.74 and 0.41±0.10, p<0.0001). Regarding PI (Fig 4a–4d), similar results were also obtained, with higher expression of Ki-67 antigen in the ALPPS than in the PVL group at 48 hours (25.65±11.34% vs. 52.42±12.80%, p<0.001), 8 days (14.46±5.98% vs. 23.73±9.11%, p<0.01) and 12 weeks (2.96±0.37 vs. 5.77±0.95, p<0.001). Interestingly, hepatic transection caused an increase in the number of Kupffer cells numbers, larger in ALPPS group, at 24 hours (28.2±3.31 vs. 39.16±8.75, p<0.01) and 8 days (59.69±8.33 vs. 75.80±8.93, p<0.0001). There were no differences about expression and distribution of CD31 antigen between groups at any time.

### Expression of HGF, TGF-β, IL6, IL1-β, TNF-α and TGF-α on the PVL and ALPPS FLR

**Fig 5:**
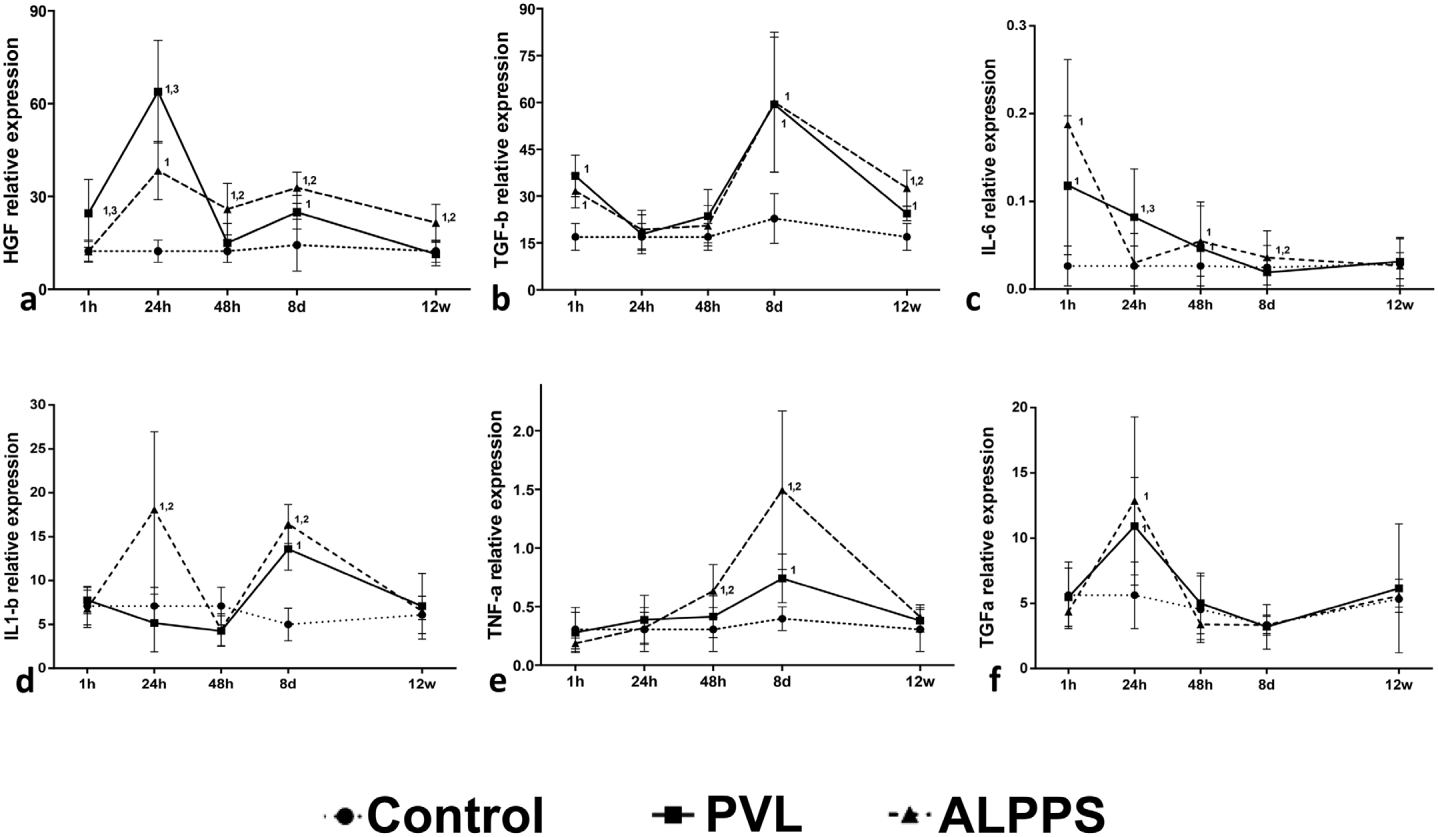
Relative expression of mRNA of (a) HGF, (b) TGF-β, (c) IL-6, (d) IL1- β, (e) TNF-α and (f) TGF-α at 1, 24, 48 hours, 8 days and 12 weeks in controls, PVL and in ALPPS FLR. Mann-Whitney U-test (n = 6 animals per group). The numbers (1, 2, 3) indicate significant differences of the considered group in comparison with control (1), PVL (2) and ALPPS (3) groups, respectively. A p value of <0.05 was considered as significant.

The molecular analysis on the FLR of both techniques revealed that the PVL achieved a larger increase in HGF than ALPPS at 1hour (p<0.005) and 24 HPS (p<0.05, Fig 5), whereas the expression of IL1-β mRNA was higher in ALPPS (p<0.05). At 48 HPS, ALPPS was associated with a higher expression of HGF and TNF-α (p<0.05) than PVL. Interestingly, at day 8 the expression of HGF, IL1-β and IL-6 was higher in ALPPS FLR (p<0.05), with a dramatic increase of TNF-α levels (p<0.05). Additionally, a high relative expression of TGF-β was observed in both groups. At week 12, levels of HGF and TGF-β were still higher on ALPPS FLR (p<0.005 and p<0.05 respectively). Expression of TGF-α was increased at 24 hours post-surgery, but there were no significant differences about its expression in both groups at this point.

### Blood and clotting investigations

**Fig 6:**
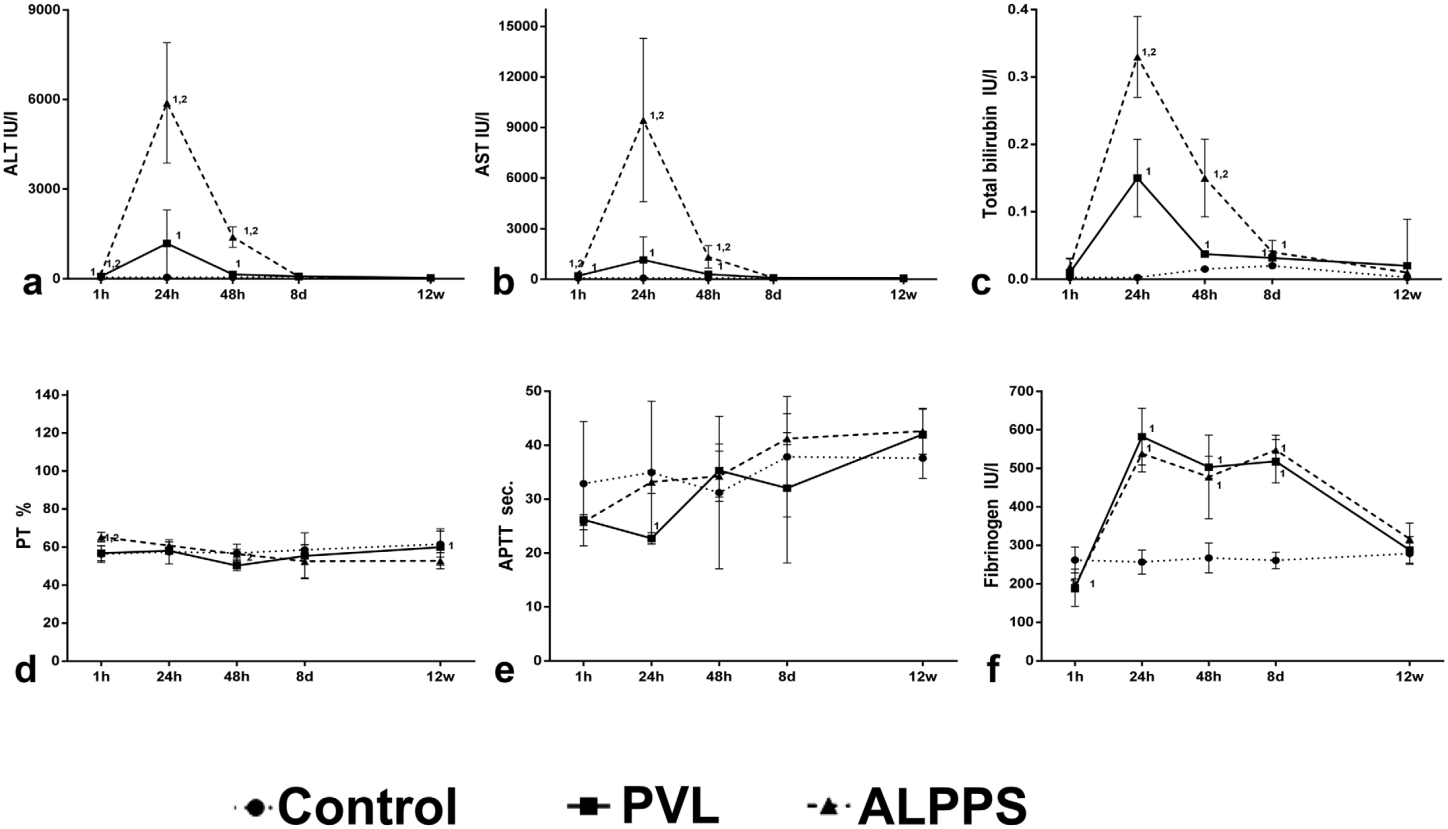
Levels of serum ALT, AST, total bilirubin, PT, APTT and fibrinogen from serum samples from control, PVL and ALPPS groups. The degree of hepatic damage induced by the first step of the ALPPS procedure was evidenced by serum levels of (**a**) ALT, (**b**) AST and (**c**) total bilirubin was significantly higher in ALPPS group in comparison with PVL. In contrast, there were no differences in (**d**) PT, (**e**) APTT and (**f**) fibrinogen levels. Mann-Whitney U test (n = 6 animals per group). The numbers (1, 2) indicate significant differences of the considered group in comparison with control (1) and PVL (2) groups, respectively. A p value of <0.05 was considered as significant.

Clinical investigations showed that the combined hepatic transection and PVL provoked severe hepatic damage, higher than the PVL alone, with a peak of serum AST, ALT and total bilirubin at 24 hours (p<0.05, Fig 6a–6c). However, interestingly enough, these levels started to decrease at 48 hours to day 8, where the AST and ALT parameters reached levels similar to those observed in controls, although total bilirubin levels were still high in both groups. Regarding clotting parameters, ALPPS led to an increase in PT at 1 hour (p<0.05, Fig 6d), but no significant changes were observed in APTT and fibrinogen levels in both groups (Fig 6e and 6f).

## Discussion

The ALPPS technique has arisen as a potential alternative in those cases in which the FLR volume is insufficient to maintain organic homeostasis after tumoral resection and enables to shorten the second step procedure avoiding the risk of tumor progression, although the exact way in which the hypertrophic stimulus is associated with the FLR is still unclear. Our point was to study establish the morphometric, molecular and clinical changes caused by the hepatic transection combined with a PVL (the first stage of the ALPPS procedure) in a microsurgery model in rats. There are several reasons for choosing the rat to develop our model: the anatomical and functional description of the rat liver is based on Couinaud´s description of the human liver [20], results are highly reproducible and the parenchymal mass of each lobe is relatively constant [17], and we gained the 2/3 of hepatic atrophic parenchyma required for an optimal regenerative response. Thus, this PVL procedure prior to proceed to the hepatic transection was deemed suitable.

Combining PVL and hepatic transection of the medium lobe led to a higher hypertrophy of the FLR, with higher MI and proliferative rate than the PVL in a similar way to the previously described partial hepatectomy model for rats [10] or mice [15], and continues in a lower degree up to 12 weeks after surgery, where the weight and volume reached by PVL FLR is similar to those observed in the ALPPS FLR at day 8. Although there are no studies in human pathology which describe the kinetics of proliferative rate on the FLR during the first steps of liver regeneration induced by the ALPPS, Schitzbauer *et al*. reported an increase in Ki-67 index on the FLR during the *in situ* split procedure [13], and it has been suggested that the growth rate of the FLR after the ALPPS procedure is similar to that of the liver after resection [14, 21], which is in line with the results from the present study. As in other studies [15], we did not found significant differences between CD31 expressions between groups.

The reason for such hypertrophy may be complex. Previous studies have pointed to the ligation/embolization of the portal vein inducing the atrophy-hypertrophy complex (AHC), a liver regenerative response following hepatocyte loss due to liver injury in which a complex, and not well understood, hemodynamics and molecular changes take place [22]. Experimental studies in rat models stated that the impairment of portal flow may be the major cause for AHC [23]. Thus, the outcome of the hyperplasia of the FLR on the two-stage hepatectomy may be based on the success of an accurate interruption of the portal flow to the atrophic lobes [6, 24], a proliferative response which appears to be independent of the loss of liver mass, as liver regenerative hyperplasia is initiated prior to the atrophy of the occluded liver [25]. Ferko *et al*. [24] observed that the presence of collateral irrigation might lead to a failure of an accurate liver hypertrophy, and Schnitzbauer *et al*. [13] suggested that the stronger proliferative response of the ALPPS technique is based on the complete devascularization of the atrophic lobe caused by the hepatic transection, which prevents the formation of vascular collaterals, and brings about a stronger hypertrophic stimulus on the FLR. Yao *et al*. [26], in a rat model of liver PVL and *in situ* splitting, observed a significant decrease in microcirculation blood perfusion and the presence of necrotic areas of the atrophic lobes in comparison with those with PVL alone. Since they did not observed differences in microcirculation blood perfusion on the FLR in both groups, they suggested that *in situ* splitting may cause higher necrotic damage to atrophic lobes that may induce a compensatory hypertrophy of the FLR. Similarly, in our model it has been observed that the hepatic transection produced a higher degree of necrosis than PVL on the atrophic lobe, but the exact complex mechanisms by which hepatic transection could trigger a higher hypertrophic response in the FLR needs to be further investigated.

For the molecular analysis, the present authors decided to analyze the expression pattern of those cytokines that are best known to be involved in hepatocyte proliferation [27]. The molecular analysis of the FLR of the ALPPS group showed several differences in the expression pattern of cytokines from the PVL. A similar expression pattern was also observed between both groups at 48 hours and 8 days. IL-6, HGF and TNF-α cytokines are well known to produce hepatocyte mitogenic stimuli during regeneration [27–30], while IL1-β has showed anti-proliferative effects on hepatocyte proliferation after hepatectomy [31–32]. Previous studies have pointed a role of IL-6 and TNF-α during the regenerative process induced by the hepatic transection [15], but no studies for the expression of other cytokines involved in hepatocyte proliferation (like IL1-β, TGF-β, TGF-α) have been yet performed. TGF-β has been properly identified as an inhibitor of mitogenic stimulus of hepatocytes [33]. Despite these observations, we observed a peak in TGF-β levels in both groups by day 8, where hepatic proliferation rates are still high. The increase in TGF-β in hepatectomy-induced liver damage had been previously observed [34], and proliferating hepatocytes seems to become refractory to the inhibitory action of TGF-β during regenerative process [31, 35]. These apparently contradictory results suggest that this cytokine may play alternative or additional roles during the first stages of liver regeneration. Thus, in the context of liver proliferation, this multifunctional molecule may play a role not only in matrix remodeling by hepatic stellate cells and promotion of angiogenesis [27, 36], but also in the expansion and differentiation of hepatic progenitor cells during liver regeneration [37]. Thus, the exact role of this cytokine during the different stages of liver regeneration still needs to be determined. Lastly, there was a similar expression pattern of TGF-α between both surgical procedures.

Overall, the increase in the proliferative stimulus observed during the first 8 days seems to be a result of a complex interaction between pro-mitogenic (IL-6, HGF and especially TNF-α) and antiproliferative (IL1-β, TGF-β) cytokines; although the expression pattern seems to be similar in both groups, the degree of the expression appears to be higher in transected livers. Liver regeneration comprises a multistep process, each of them characterized by the expression and secretion of transcription factors and cytokines [38–39] in which the hepatocytes are proliferating for 12–72 hours after the priming phase [27]. According to this, the cytokine expression pattern observed in our study is similar to those previously described for hepatectomy models but, in contrast, it seems that the proliferative phase of the cycle induced by PVL/ALPPS is extended to 8 days after surgery. Although the exact source of all these cytokines could not be identified, an increase in Kupffer cell numbers in the ALPPS FLR was observed, which are known to be the main source of IL1-β and TNF-α during the regenerative process [40]. Despite the fact that macrophages act as these cytokine promoters in liver regeneration, there is growing evidence that macrophages can also promote the hepatic progenitor cell niche following hepatocellular injury by engulfment hepatocyte debris and liberation of promoters of activation of hepatic progenitor cells [41–42].

One of the limitations of this study lies in the impossibility to perform the split technique in all the hepatic lobes, and the hypertrophic and atrophic stimuli in rat liver seem to be more pronounced than in humans [43]. Another point is that several groups have pointed that the portal vein ligation does not lead to excessive hepatic necrosis in rats because the liver-specific differentiation and function is still preserved, and might help to maintain homeostasis during the fast regeneration of the FLR minimizing any significant elevation in liver enzymes [5, 20, 24]. Nevertheless, despite these features, it has been observed that the transection of the medium lobe leads to similar morphometric effects to its human counterpart.

As some authors suggest that the increase in proliferative rate associated with ALPPS is not necessarily reflected in its functional capability [14], it was decided to study the effect on the FLR after the second stage of the ALPPS procedure within 12 weeks after surgery. At this time, no significant histopathologic feature of the parenchymal architecture in the FLR of both groups was observed, and the clinical analysis revealed no alterations, although there was still a stronger proliferative stimulus in ALPPS FLR than in PVL. Our data points to maintained proliferation stimuli in which the HGF and TGF-β cytokines seem to play a role, but the exact way in which the transection procedure brings about this higher long-term proliferative stimulus still needs to be determined.

Although apparently there is a substantial benefit on the application of the ALPPS in terms of morphometric and proliferative rate, previous studies in human pathology observed that, in contrast with the PVL or PVE techniques, this procedure brings about high rates of morbidity and mortality, which calls into question the safety and clinical applicability of this technique [8,44–47]. Our clinical data and mortality rate demonstrates that the split-off of the median lobe causes a severe liver damage, substantially higher than the PVL alone during the first 48 hours. After having reached this peak, clinical parameters gradually came back to normal values until day 8. Thus, it seems that the first 48 hours after surgery are critical for survival in our model, but it is important to consider that all of the experiences have been performed on healthy animals, a fact that should be taken into account on its application in human medicine, in which the liver parenchyma proliferation rate is usually altered by the chemotherapy treatment or by the disease itself.

## Conclusions

Overall, the hepatic transection combined with PVL caused a higher hypertrophic response than the PVL alone with a different molecular expression pattern. Further studies are necessary to determine the complex interactions which lead to such proliferation, but the use of this experimental model in rats may not only help understand these features, but also be a valuable tool for oncologic research. In this sense, there are very recent reports that mention that ALPPS might be associated with a high rate of hepatic and extra hepatic recurrence at long-term examination [42]. The way in which the extreme hypertrophy caused by the ALPPS procedure promotes tumor recurrence remains uncertain and needs to be determined. The model here presented will certainly help solve this and many other issues in order to optimize the ALPPS technique by means of effectiveness and waiting time until satisfactory hypertrophy of the FLR takes place.

## References

[1] Adam R, Laurent A, Azoulay D, Castaing D, Bismuth H. Two-stage hepatectomy: a planned strategy to treat irresectable liver tumors. Ann Surg 2000; 6:777–85 10.1097/00000658-200012000-00006 PMC1421270 11088072

[2] Clavien PA, Petrowsky H, DeOliveira ML, Graf R. Strategies for safer liver surgery and partial liver transplantation. N Engl J Med 2007; 356:1545–59. 17429086 10.1056/NEJMra065156

[3] Tanaka K, Shimada H, Matsuo K, Ueda M, Endo I, Togo S. Regeneration after two-stage hepatectomy vs. repeat resection for colorectal metastasis recurrence. J. Gastrointest Surg 2007; 11:1154–61. 17623261 10.1007/s11605-007-0221-0

[4] Broering DC, Hillert C, Krupski G, Fischer L, Mueller L, Achilles EG, et al Portal vein embolization vs. portal vein ligation for induction of hypertrophy of the future liver remnant. J Gastrointest Surg 2002; 6:905–13. 12504230 10.1016/s1091-255x(02)00122-1

[5] Wilms C, Mueller L, Lenk C, Wittkugel O, Helmke K, Krupski-Berdien G, et al Comparative study of portal vein embolization versus portal vein ligation for induction of hypertrophy of the future liver remnant using a mini-pig model. Ann Surg 2008; 247:825–34. 10.1097/SLA.0b013e31816a9d7c 18438120

[6] Azoulay D, Castaing D, Smail A, Adam R, Cailliez V, Laurent A, et al Resection of nonresectable liver metastases from colorectal cancer after percutaneous vein embolization. Ann Surg 2000; 231:480–6. 10749607 10.1097/00000658-200004000-00005 PMC1421022

[7] Kokudo N, Tada K, Seki M, Ohta H, Yamaguchi T, Matsubara T, et al Proliferative activity of intrahepatic colorectal metastases after preoperative hemihepatic portal vein embolization. Hepatology. 2001; 34:267–72. 11481611 10.1053/jhep.2001.26513

[8] Heinrich S, Lang H. Liver metastases from colorectal cancer: technique of liver resection. J Surg Oncol 2013; 107:579–84. 10.1002/jso.23138 22566374

[9] Aussilhou B, Lesurtel M, Sauvanet A, Farges O, Dokmak S, Goasguen N, et al Right portal vein ligation is as efficient as portal vein embolization to induce hypertrophy of the left liver remnant. J Gastrointest Surg 2008; 12:297–303. 18060468 10.1007/s11605-007-0410-x

[10] Furrer K, Tian Y, Pfammatter T, Jochum W, El-Badry AM, Graf R, et al Selective portal vein embolization and ligation trigger different regenerative responses in the rat liver. Hepatology 2008; 47:1615–23. 10.1002/hep.22164 18395841

[11] Tashiro S. Mechanism of liver regeneration after liver resection and portal vein embolization (ligation) is different?. Hepatobiliary Pancreat Surg 2009; 16:292–9. 10.1007/s00534-009-0058-x 19333540

[12] de Santibanes E, Clavien PA. Playing Play-Doh to prevent postoperative liver faliure: the “ALPPS” approach. Ann Surg 2012; 255:415–7. 10.1097/SLA.0b013e318248577d 22330039

[13] Schnitzbauer AA, Lang SA, Goessmann H, Nadalin S, Baumgart J, Farkas SA, et al Right portal vein ligation combined with in situ splitting induces rapid left lateral liver lobe hypertrophy enabling 2-staged extended right hepatic resection in small-for-size settings. Ann Surg 2012; 255:405–14. 10.1097/SLA.0b013e31824856f5 22330038

[14] Tschuor CH, Croome KP, Sergeant G, Cano V, Schadde E, Ardiles V, et al Salvage parenchymal liver transection for patients with insufficient volume increase after portal vein occlusion—an extension of the ALPPS approach. Eur J Surg Oncol 2013; 39:1230–5. 10.1016/j.ejso.2013.08.009 23994139

[15] Schlegel A, Lesurtel M, Melloul E, Limani P, Tschuor C, Graf R, et al ALPPS: from human to mice highlighting accelerated and novel mechanisms of liver regeneration. Ann Surg 2014; 260:839–847. 10.1097/SLA.0000000000000949 25379855

[16] Rozga J, Jeppson B, Bengmark S. Portal branch ligation in the rat. Reevaluation of a model. Am J Pathol 1986; 125:300–8. 3789089 PMC1888241

[17] Madrahimov M, Dirsch O, Broelsch C, Dahmen U. Marginal hepatectomy in the rat. From anatomy to surgery. Ann Surg 2006; 244:89–98. 16794393 10.1097/01.sla.0000218093.12408.0f PMC1570604

[18] Abraldes JG, Pasarín M, García-Pagán JC. Animal models of portal hypertension. World J Gastroenterol 2006; 12:6577–84. 17075968 10.3748/wjg.v12.i41.6577 PMC4125660

[19] Niehues SM, Unger JK, Malinowski M, Neymeyer J, Hamm B, Stockmann M. Liver volume measurement: reason of the difference between in vivo CT-volumetry and intraoperative ex vivo determination and how to cope it. Eur J Med Res 2010; 15:345–50. 20947471 10.1186/2047-783X-15-8-345 PMC3458704

[20] Aller MA, Arias N, Prieto I, Agudo S, Gilsanz C, Lorente L, et al A half century (1961–2011) of applying microsurgery to experimental liver research. World J Hepatol 2012; 4:199–208. 10.4254/wjh.v4.i7.199 22855695 PMC3409354

[21] Sotiropoulos GC, Kouraklis G. The ALPPS procedure for extended indications in liver surgery: an old finding applied in surgical oncology. Ann Surg 2013; 257:e26 10.1097/SLA.0b013e3182942e4a 23629529

[22] Kim RD, Kim JS, Watanabe G, Mohuczy D, Behrns KE. Liver regeneration and the atrophy-hypertrophy complex. Semin Intervent Radiol 2008; 25:92–103. 10.1055/s-2008-1076679 21326550 PMC3036484

[23] Schweizer W, Duda P, Tanner S, Balsiger D, Höflin F, Blumgart LH, et al Experimental atrophy/hypertrophy complex (AHC) of the liver: portal vein, but not bile duct obstruction, is the main driving force for the development of AHC in the rat. J Hepatol 1995; 23:71–8. 8530812 10.1016/0168-8278(95)80313-0

[24] Ferko A, Lesko M, Krajina A, Hulek P, Rösch J, Rabkin J. Intrahepatic portal vein branches after extrahepatic portal vein occlusion. Experimental study. Hepatogastroenterology 2001; 48:475–9. 11379337

[25] Lambotte L, Li B, Leclerq I, Sempoux C, Saliez A, Horsmans Y. The compensatory hyperplasia (liver regeneration) following ligation of a portal branch is initiated before the atrophy of the deprived lobes. J Hepatol 2000; 32:940–5. 10898314 10.1016/s0168-8278(00)80098-7

[26] Yao L, Li C, Ge X, Wang H, Xu K, Zhang A, et al Establishment of a rat model of portal vein ligation combined with *in situ* splitting. PLOS one 2014; 9:e105511 10.1371/journal.pone.0105511 25144490 PMC4140771

[27] Michalopoulos GK. Liver regeneration. J Cell Physiol 2007; 213:286–300. 17559071 10.1002/jcp.21172 PMC2701258

[28] Diehl AM, Yin M, Fleckenstein J, Yang SQ, Li HZ, Brenner DA, et al Tumor necrosis factor-alpha induces c-jun during the regenerative response to liver injury. Am J Physiol 1994; 267:G552–61. 7943321 10.1152/ajpgi.1994.267.4.G552

[29] Iocca HA, Isom HC. Tumor necrosis factor-alpha acts as a complete mitogen for primary rat hepatocytes. Am J Pathol 2003; 163:465–76. 12875968 10.1016/s0002-9440(10)63676-0 PMC1868193

[30] Liska V, Treska V, Mirka H, Kobr J, Sykora R, Skalicky T, et al Tumor necrosis factor-alpha stimulates liver regeneration in porcine model of partial vein ligation. Hepatogastroenterology 2012; 59:496–500. 10.5754/hge10265 22353515

[31] Boulton R, Woodman A, Calnan D, Selden C, Tam F, Hodgson H. Nonparenchymal cells from regenerating liver generate interleukin1-α and 1-β: a mechanism of negative regulation of hepatocyte proliferation. Hepatology 1997; 26:49–58. 9214451 10.1053/jhep.1997.v26.pm0009214451

[32] Ogiso T, Nagaki M, Takai S, Tsukada Y, Mukai T, Kimura K, et al Granulocyte colony-stimulating factor impairs liver regeneration in mice through the up-regulation of interleukin-1β. J Hepatol 2007; 47:816–25. 17869372 10.1016/j.jhep.2007.06.017

[33] Zhong Z, Tsukada S, Rehman H, Parsons CJ, Theruvath TP, Rippe RA, et al Inhibition of transforming growth factor-β/Smad signaling improves regeneration of small-for-size rat liver grafts. Liver Transpl 2010; 16:181–90. 10.1002/lt.21966 20104486 PMC2834418

[34] Braun L, Mead JE, Panzica M, Mikumo R, Bell G, Fausto N. Transforming growth factor β mRNA increases during liver regeneration: a possible paracrine mechanism of liver regeneration. PNAS 1988; 85:1539–43. 3422749 10.1073/pnas.85.5.1539 PMC279808

[35] Chari RS, Price DT, Sue SR, Meyers WC, Jirtle RL. Down-regulation of transforming growth factor beta receptor type I, II and III during liver regeneration. Am J Surg 1995; 169:126–31. 7817981 10.1016/s0002-9610(99)80120-2

[36] Nakatsukasa H, Evarts RP, Hsia CC, Thorgiersson SS. Transforming growth factor β_1_ and type I procollagen transcripts during regeneration and early fibrosis of rat liver. Lab Invest 1990; 63:171–80. 2381163

[37] Thenappan A, Li Y, Kitisin K, Rashid A, Shetty K, Johnson L, et al. Role of transforming growth factor β signaling and expansion of progenitor cells in regenerating liver. Hepatology 2010; 51:1373–82. 10.1002/hep.23449 20131405 PMC3001243

[38] Fausto N. Liver regeneration. J Hepatol 2000; 32 (1 Suppl):19–31. 10728791 10.1016/s0168-8278(00)80412-2

[39] Kountouras J, Boura P, Lygidakis NJ. Liver regeneration after hepatectomy. Hepatogastroenterology 2001; 48:556–62. 11379353

[40] Diehl AM, Rai RM. Liver regeneration 3: Regulation of signal transduction during liver regeneration. FASEB J 1996; 10:215–27. 8641555 10.1096/fasebj.10.2.8641555

[41] Boulter L, Govaere O, Bird TG, Radulescu S, Ramachandran P, Pellicoro A, et al Macrophage-derived Wnt opposes Notch signaling to specify hepatic progenitor cell fate in chronic liver disease. Nat Med 2012; 18:572–9. 10.1038/nm.2667 22388089 PMC3364717

[42] Bird TG, Boutler L, Cole A, Lorenzini S, Lu WY, Hay T, et al Manipulation of liver regeneration with macrophages to influence the hepatic progenitor cell niche. Lancet 2013; 381:S23.

[43] Mueller L, Grotelueschen R, Meyer J, Vashist YK, Abdulgawad A, Wilms C, et al Sustained function in atrophying liver tissue after portal branch ligation in the rat. J Surg Res 2003; 114:146–55. 14559440 10.1016/s0022-4804(03)00252-x

[44] Schadde E, Ardiles V, Slankamenac K, Tschuor C, Sergeant G, Amacker N, et al ALPPS offers a better chance of complete resection in patients with primarily unresectableliver tumors compared with conventional-staged hepatectomies: results of a multicenter analysis. World J Surg 2014; 38:1510–9. 10.1007/s00268-014-2513-3 24748319

[45] Figueras J, Belguiti J. The ALPPS approach: should we sacrifice basic therapeutic rules in the name of innovation?. World J Surg 2014; 38: 1520–1. 10.1007/s00268-014-2540-0 24756547

[46] Abdalla EK, Hicks ME, Vauthey JN. Portal vein embolization: rationale, technique and future prospects. Br J Surg 2001; 88:165–75. 11167863 10.1046/j.1365-2168.2001.01658.x

[47] Oldhafer KJ, Donati M, Jenner RM, Stang A, Stavrou GA. ALPPS for patients with colorectal liver metastases: effective liver hypertrophy, but early tumor recurrence. World J Surg 2014; 38:1504 **–** 9. 10.1007/s00268-013-2401-2 24326456

